# Disentangling the links between habitat complexity and biodiversity in a kelp‐dominated subantarctic community

**DOI:** 10.1002/ece3.7100

**Published:** 2021-01-15

**Authors:** Catalina Velasco‐Charpentier, Felipe Pizarro‐Mora, Nelso P. Navarro, Nelson Valdivia

**Affiliations:** ^1^ Centro de Investigación Gaia Antártica Universidad de Magallanes Punta Arenas Chile; ^2^ Fundación Mar y Ciencia Punta Arenas Chile; ^3^ Laboratorio de Ecofisiología y Biotecnología de Algas (LEBA) Facultad de Ciencias Universidad de Magallanes Punta Arenas Chile; ^4^ Centro FONDAP de Investigación Dinámica de Ecosistemas Marinos de Altas Latitudes (IDEAL) Universidad Austral de Chile Valdivia Chile; ^5^ Instituto de Ciencias Marinas y Limnológicas Facultad de Ciencias Universidad Austral de Chile Valdivia Chile

**Keywords:** benthic communities, biodiversity, habitat complexity, Kelp forest, Patagonia

## Abstract

Habitat complexity is one of the most important factors modulating species diversity. This feature comprises several interrelated attributes, such as number, size, and spatial arrangement of complexity‐forming elements. However, the separate and joint effects of these attributes on diversity and community structure are still not well understood. Here, we assess the relationships between several structural‐complexity attributes of the subantarctic kelp *Lessonia flavicans* and species richness, total abundance, and structure of kelp‐associated macrobenthic communities. We predicted that longer thalli and larger holdfasts favor greater species richness and total abundance of invertebrate organisms. To test the prediction, an observational sampling program was established in two sites of the Strait of Magellan. Uni‐ and multivariate analyses revealed both positive and negative effects of kelp structural‐complexity attributes on diversity. Holdfast diameter and maximum frond length, followed by thallus wet weight, had the strongest positive fits to species richness and total abundance; the number of stipes, on the other hand, was negatively associated with both response variables. Longer fronds were associated with greater abundances of spirorbid polychaetes. Larger holdfasts supported larger abundances of Nereididae and Terebelidae polychaetes and the limpet *Nacella mytilina*. Contrarily, kelps with longer fronds and more stipes supported fewer amphipods. In this way, we demonstrate that different dimensions of habitat complexity can have contrasting effects on diversity and community structure, highlighting the fundamental role of multiple dimensions of kelp habitat complexity for local biodiversity.

## Graphical abstract

We demonstrate that different dimensions of habitat complexity can have contrasting effects on diversity and community structure. Our study highlights the fundamental role of the multiple dimensions of kelp habitat complexity for local biodiversity as a balance between positive and negative effects on associated species.

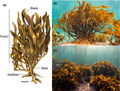

## 1 INTRODUCTION

Species diversity, or biodiversity, is perhaps the most striking feature of our biosphere. However, the unprecedent anthropogenic climate crisis poses severe threats to biodiversity across scales and realms (Trisos et al., 2020). Anthropogenic impacts on biodiversity can be particularly severe when affecting species with special functional traits, such as foundation species. Kelps, mussels, and coral are examples of foundation species that support local biodiversity and define entire ecological communities by enhancing habitat's physical complexity (e.g., Ellison et al., 2005). The loss of foundation species results therefore in habitat destruction and simplification, one of the main drivers of community alteration worldwide (Luypaert et al., 2020). However, our understanding of the relationship between species diversity and the structural complexity of biogenic habitats is still incipient (Miller et al., 2018).

Since the first publication of the effects of habitat complexity on species diversity (MacArthur & MacArthur, 1961), many studies have evinced a positive relationship between the structural complexity of habitats and species diversity (Dean & Connell, 1987; Loke & Todd, 2016; Luckhurst & Luckhurst, 1978). The mechanisms underpinning this relation include, but are not limited to, an increasing number of niches due to increased microhabitat availability, higher food web productivity and stability, and enhanced protection from physical disturbances (reviewed by Kovalenko et al., 2012). However, the use of multiple definitions of complexity has limited our understanding of its role in influencing species diversity (Kovalenko et al., 2012). For example, habitat complexity can be described as the variation in the size of the living spaces and structural components (Loke & Todd, 2016), or just as the number of structural components of the habitat (known as “heterogeneity”; McCoy & Bell, 1991).

Habitat complexity is a multifaceted concept, encompassing five interrelated dimensions (Tokeshi & Arakaki, 2012): scale of observation of the structural elements (e.g., global, regional, local, or microhabitat); diversity of elements (i.e., surface geometry or topography); spatial arrangement of elements (the elements may be scattered randomly, clustered, in patches of different elements, or in zonation); size of the structural elements; and their density (i.e., number of elements in a determined area). These criteria provide a more accurate view of how habitat complexity is related to species diversity and community structure. Within a given habitat, for example, a greater size range of structural elements can support a greater species diversity and abundance due to enhanced niche availability (e.g., Hacker & Steneck, 1990; Loke & Todd, 2016; Tsuchiya & Nishihira, 1986).

**FIGURE 1:**
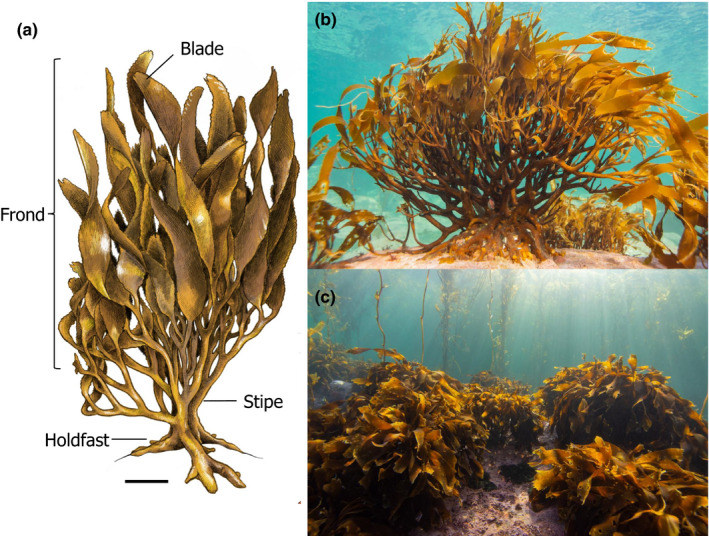
Kelp *Lessonia flavicans* in the Strait of Magellan. Scheme (a) and photograph (b) depicting the sections of the kelp (frond, stipe, and holdfast). *Lessonia flavicans* belt‐shaped kelp forest in Bahía Buzos at 2 m depth (c). The scale bar in panel (a) equals 10 cm. Ilustration credit: Martin Mak; Photo credits: Catalina Velasco‐Charpentier

Kelp forests are large brown macroalgae that support many temperate coastal marine habitats (Mora‐Soto et al., 2020; Smale, 2019). As foundation species, mature thalli comprise multiple habitat‐complexity attributes, such as number of stipes, total height, holdfast diameter, foliage cover, and kelp density (Dayton, 1985; Figure 1). Kelp forests enhance local species diversity through increasing primary productivity, nutrient supply, shelter, and secondary settlement space (Arkema et al., 2009; Duggins & Eckman, 1997; Steneck et al., 2002). Despite their important role as complex habitats, kelp's complexity attributes are usually analyzed separately (Villouta & Santelices, 1984; Vega et al., 2014), and most studies focus on complexity without a clear distinction of attributes (but see Trujillo et al., 2019). So, little is known about which complexity attribute(s) are playing the most relevant role in determining how the kelp‐associated community is structured.

The kelp *Lessonia* spp. have major ecological roles structuring benthic marine communities on Chilean temperate coasts (Vásquez & Santelices, 1984; Villouta & Santelices, 1984). The available information about this genus in the Chilean Pacific coast is mainly based on studies of *Lessonia berteroana* and *L. spicata* in northern and northern‐central Chile (Vásquez & Santelices, 1984; Vásquez et al., 2012; Vega et al., 2014; Vega, 2016). *Lessonia* species are currently under strong harvesting pressure for the extraction of alginic acid in these latitudes (Steneck et al., 2002). Illegal fishermen use pry bars to detach the entire kelps from the substratum, an activity that threatens to move southward soon (Rosenfeld et al., 2019). Yet, the functional roles of this group are largely unknown in southern cold‐temperate and subpolar regions, where *Lessonia flavicans* (Bory 1825) is one of the most conspicuous macroalga (Mansilla et al., 2014; Marambio et al., 2016).

In this study, we assess the relationships between several structural‐complexity attributes of *L. flavicans* and species richness, total abundance, and structure of kelp‐associated macrobenthic invertebrate communities. We hypothesized that longer fronds and larger holdfasts will favor greater species richness and total abundance due to enhanced secondary settlement space. These effects should be reflected in strong associations between frond size, holdfast diameter, and community structure (i.e., the combination of species identities and abundances).

## 2 MATERIALS AND METHODS

### 2.1 Study sites and sampling design

The Magellan region is located in the South East Pacific, encompassing a diversity of habitats that include fjords, inland seas, glaciers, gulfs, and channels—a complex landscape resulting from the combined effect of tectonic processes and glaciation. The oceanographic features and diverse environmental conditions determine a particular marine biogeographic unit (Camus, 2001) dominated by benthic invertebrates and extensive kelp forests (Friedlander et al., 2018).

**FIGURE 2:**
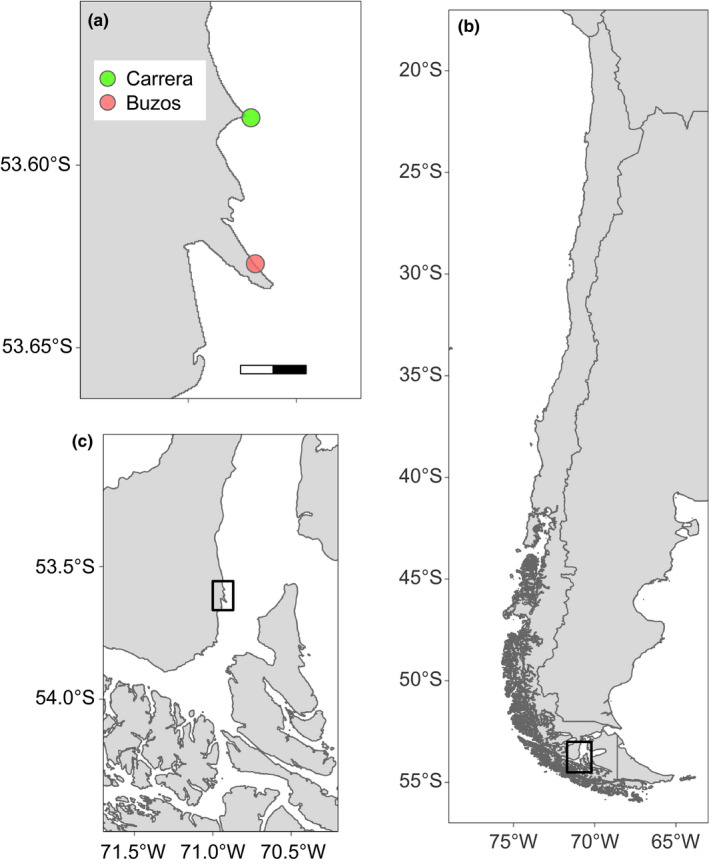
Map of the sampling sites, Bahía Buzos and Carrera (a), located in the Strait of Magellan (b), Chile (c)

To analyze the potential spatial variation in the effects of kelp's structural complexity, the study was conducted at two sites (4 km apart) in the Strait of Magellan (Figure 2), named “Bahía Buzos” (−53.627°S, −70.919°W) and “Carrera” (−53.587°S, −70.921°W). Both sites harbor *L. flavicans* forests of around 400 m^2^, located between 0.5 and 4 m depth (C. Velasco‐Charpentier pers. obs.). Both sites are moderately wave‐exposed, with a hard substratum consisting of boulders and large rocks covered by crustose coralline algae. The patchy rocky reefs extend from the intertidal to ~8 m depth.

An observational sampling program of kelp habitat complexity and associated communities was established in both sites between November 2018 and August 2019. Observations were conducted every three months in both sites. Punta Carrera was sampled only during spring and summer due to logistics limitations and harsh weather. At each sampling time and site, SCUBA divers deployed a 100 m transect parallel to the coastline. The transects were placed on substrata of similar rock composition (boulders), depth (0.5–2 m), and inclination (~0°). In each transect, ten 0.25 m^2^ quadrants separated by ~3 m were photographed with a Nikon D7200 camera equipped with an Ikelite housing and a rectilinear Tokina 11–16 lens. The unit of replication was each kelp (*N* = 10 per site and season) since we selected the quadrants with one *L. flavicans* specimen. Each thallus was wrapped with a mesh bag (1 mm^2^ pore size). Then, the holdfast was detached from the rock with a knife and the bag was swiftly closed to minimize the escape of mobile macrobenthic organisms. Within one hour after collection, samples were transported to the Laboratorio de Ecofisiología y Biotecnología de Algas, Universidad de Magallanes to measure the thallus morphology attributes and species sorting.

### 2.2 Estimation of kelp structural complexity and associated biodiversity

Once in the laboratory, we measured five kelp structural‐complexity attributes. The number of stipes, maximum length (i.e., thallus length, from holdfast base to the apex in cm), holdfast diameter (i.e., maximum width of the holdfast base in cm), and total wet weight in g (0.01 g precision) were directly measured from the collected kelps. Foliage cover was estimated from each digital photograph taken in situ from above, at a zenith angle. This variable was categorized as 1 (fronds cover between <10 and 30% of the quadrant), 2 (fronds cover between 31% and 50%), 3 (fronds cover between 51% and 80%), and 4 (frond cover between 81% and 100%).

For each *L. flavicans* thallus, macrobenthic (>1 mm) mobile and semi‐sessile organisms (e.g., mussels) in addition to tube‐dwelling sessile organisms (e.g., spirorbid polychaetes) were collected from thallus surface with aid of forceps and scalpels. In addition, the stipes and disks were dissected to collect organisms living in crevices and galleries. We used specialized literature and field guides to identify each individual to the lowest taxonomic level possible, usually species (González‐Wevar et al., 2018; Häussermann & Försterra, 2009; McLean, 1984; Osorio et al., 1979; Pastorino, 2005). Species‐specific abundance was estimated as counts of individuals. These data were used to estimate species richness, defined as the number of taxonomic identities; total abundance, defined as the total number of individuals per taxonomic identities; and community structure, defined as the combination of species identities and abundances. Before the analyses, Pearson‐product moment correlations were calculated among the explanatory variables in order to account for collinearity.

### 2.3 Univariate analyses

Generalized linear models (GLM) were used to analyze species richness and total abundance separately. Since the explanatory variables (number of stipes, maximum length, holdfast diameter, total wet weight, and foliage cover) were highly correlated (Figure S1), we first combined these variables in a principal component analysis (PCA). In the GLMs, the explanatory variables were the PC scores of the first two axes (64% of explained variance), in addition to season and site. Since one site was sampled only in two seasons, we did not include the site by season interactive effect in the models. Thus, the sites were compared by averaging the seasons, while the seasons were compared by averaging both sites. For each categorical variable, we used a “treatment” contrast in which the mean value of each group is compared against a reference group: Bahía Buzos was the reference site because was the site with more sampling events, while spring was the reference for season because it is usually the season with larger species abundances. The use of this contrast type allowed us to keep the number of comparisons below the number of groups in the case of season and thus to prevent the inflation of Type‐I error type.

Due to the difference in measurement scales, the continuous response variables were centered and standardized before the analyses (Becker et al., 1988). Model parameters were estimated through maximum likelihood. For both models, that is, species richness and total abundance, we assumed a Poisson distribution of errors and we estimated a marginal coefficient of determination (*R* ^2^) according to Nakagawa and Schielzeth (2013).

### 2.4 Multivariate analyses

Multivariate community structure was analyzed with canonical analysis on principal coordinates (CAP, Anderson & Willis, 2003). CAP is a constrained multivariate method in which an a priori prediction (e.g., maximum length and holdfast diameter strongly relate with community structure) is used to produce an ordination plot. The CAP axes are linear combinations of the response variables that maximize the between‐ to within‐group variation, allowing the detection of patterns that could be masked by overall dispersion in unconstrained methods (Anderson & Willis, 2003). The model was based on species abundances, which were used to estimate a matrix of Bray–Curtis dissimilarities. In addition to kelp's structural‐complexity attributes, season and site were included as fixed effects in the model. The statistical significance of the CAP model fit was assessed by means of an analysis of variance.

All analyses were conducted in the R statistical environment version 4.0.2 (R Core Team, 2019). We used tidyverse (Wickham et al., 2019), vegan (Oksanen et al., 2019), lme4 (Bates et al., 2015), sjPlot (Lüdecke, 2020), MuMIn (Barton, 2020), and cowplot (Wilke, 2019) R packages to generate graphs and compute descriptive statistics and models.

## 3 RESULTS

**FIGURE 3:**
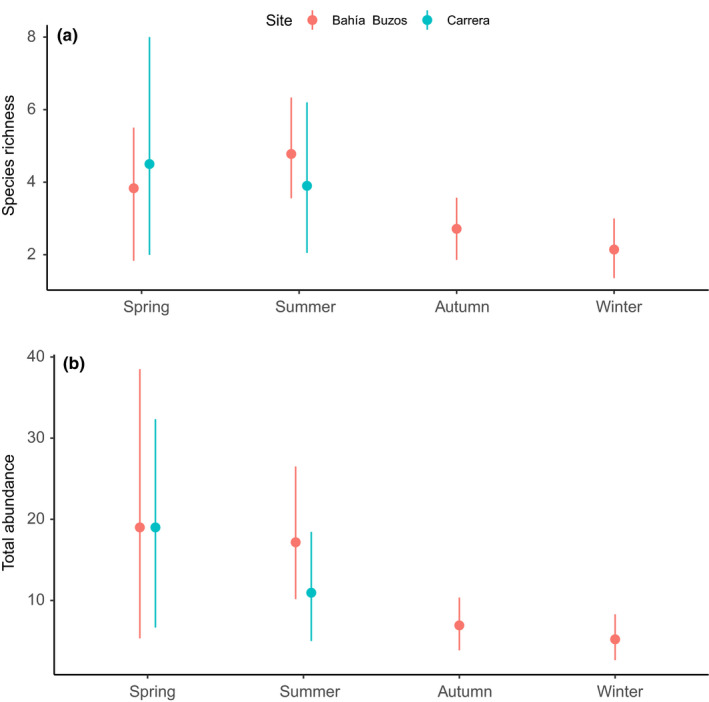
Species richness (a) and total abundance (b) of mobile organisms associated with kelp *Lessonia flavicans* across austral seasons (November 2018–August 2019) and two sites, Bahía Buzos and Carrera, in the Strait of Magellan. ND, no data. Points represent the mean values (*N* = 10 kelps), while bars represent 95% confidence intervals (CI)

A total of 41 taxonomic identities were identified associated with *L. flavicans* kelps across both sites (Table 1). Mollusca, with 18 species, was the most diverse phylum. The most abundant species were the isopod *Cassidinopsis emarginata* (*N* = 186; on kelp fronds), the amphipod *Peramphithoe femorata* (*N* = 107; on kelp fronds), and polychaetes of the Terebellidae family (*N* = 184; in kelp holdfasts). The bivalves *Aulacomya atra* and *Mytilus chilensis* were always found in juvenile stages (<4 cm), in crevices and galleries formed in the kelp's holdfasts. Species richness and total abundance tended to increase in spring and summer periods (Figure 3)—both variables were greater in spring‐summer than winter‐fall. All kelp habitat complexity dimensions were positive and significantly correlated, except the number of stipes and wet weight, which were uncorrelated. The strongest correlation was observed between holdfast diameter and wet weight (Figure S1).

**TABLE 1:** List of mobile and semi‐sessile species found in each kelp and differentiated in microhabitats of *Lessonia flavicans* thallus. Organisms are shown as total abundance

Taxonomic identity	Microhabitat	
**Mollusca**	Frond	Holdfast
*Aulacomya atra*	0	40
*Mytilus chilensis*	0	25
*Hiatella arctica*	0	5
*Nacella deaurata*	3	10
*Nacella flamea*	3	3
*Nacella mytilina*	71	0
*Margarella violacea*	5	0
*Trophon geversianus*	0	1
*Acanthina monodon*	0	3
*Eumetula pulla*	0	5
*Xymenopsis (cf) muriciformis*	0	3
*Fissurella picta*	2	27
*Fisurella oriens*	0	4
*Plaxiphora aurata*	1	4
*Tonicia* sp1	1	1
*Tonicia* sp2	0	1
*Crepipatella (cf) dilatata*	0	1
*Itaxia falklandica*	2	1
**Crustacea**		
*Halicarcinus planatus*	2	12
*Cassidinopsis emarginata*	179	7
*Peramphithoe femorata*	105	2
Ampithoidae sp1	12	0
Sphaeromatidae	0	17
*Eurypodius latreillei*	2	0
*Pagurus* sp.	0	1
**Echinodermata**		
*Anasterias antarctica*	6	17
*Odontaster penicilatus*	0	5
Asteroidea sp1	1	3
*Loxechinus albus*	2	1
*Pseudechinus magellanicus*	1	34
*Arbacia dufresnii*	0	4
*Ophiomyxa vivipara*	0	25
*Ophiactic asperula*	0	24
*Psolus patagonicus*	0	17
**Cnidaria**		
*Antholoba acates*	0	5
**Annelida**		
Nereididae	5	113
Terebellidae	3	51
*Chaetopterus variopedatus*	0	14
*Spirorbis* sp.	5	31
**Nemertina**		
Nemertea sp1	0	11
**Chordata**		
Osteichthyes sp1	1	0

**FIGURE 4:**
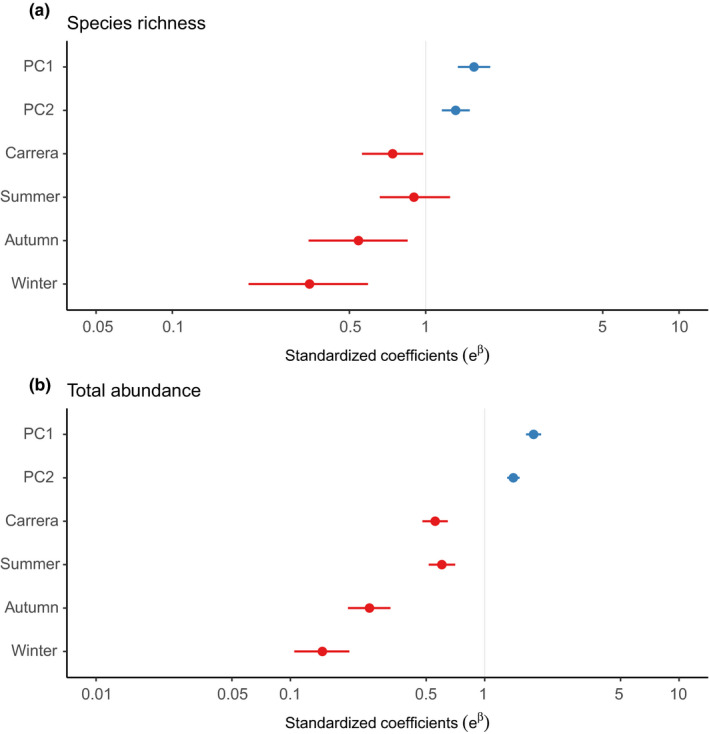
Standardized coefficients of generalized linear models including PCA axis, season and site for species richness (a) and total abundance (b) of kelp‐associated communities. Points represent the model parameters, while bars represent confidence intervals (CI). Red and blue symbols represent negative and positive standardized coefficients, respectively. CIs that do not cross 1 indicate statistically significant parameters. *Pairwise contrasts between sites (Carrera and Bahía Buzos) and between each year season and spring. The outputs are back transformed from the original log link (*e* = number of Euler, *β* = coefficient in log scale)

The principal component analysis exhibited that holdfast diameter and maximum length had the strongest positive influence on PC1, while wet weight (positive) and number of stipes (negative) had the strongest influence on PC2 (Table 2). In the GLMs, both principal components were positively related to species richness (Figure 4a, pseudo‐*R^2^* = 0.51) and total abundance (Figure 4b, pseudo‐*R^2^* = 0.88; see also Figure S2). Nevertheless, the effect of the PC1 on both response variables was larger than that of PC2 (Figure 4, Figure S2). These results demonstrate that both, species richness and total abundance, were higher in the kelps with a greater holdfast diameter, maximum length, wet weight, but with fewer stipes.

**TABLE 2:** Loadings for the first (PC1) and second (PC2) principal components axes for five kelp habitat complexity attributes

	PC1	PC2
Wet weight	0.44	**0.56**
Foliage cover	0.43	0.12
Maximum length	**0.47**	−0.29
Number of stipes	0.36	**−0.73**
Holdfast diameter	**0.50**	0.19

Note

The first and second PC axis explained 64% and 19% of the variance, respectively. PC loadings with absolute values > 1/√5 are in bold.

The site Bahía Buzos, on average, presented a greater species richness and total abundance than Carrera (Figure 4a,b, respectively; Figure S2). Regarding seasonality, species richness and total abundance decreased from spring to winter, which was represented by increasingly negative effect coefficients (Figure 4a,b, respectively; Figure S2). However, the difference in mean species richness between summer and spring was negligible (Figure 4a, Figure S2).

**FIGURE 5:**
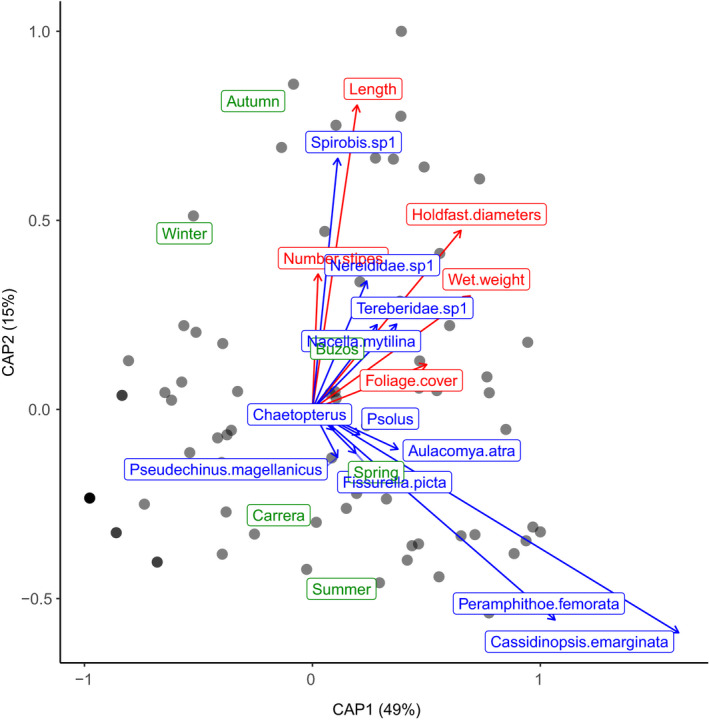
Canonical analysis of principal coordinates for community structure across quadrants. Red is for the kelp complexity attributes, green for sites and year seasons, and blue is for the associated species. Only species with |standardized scores| > 0.05 are shown to reduce distortions in the ordination. Darker dots indicate overlapping observations

The canonical analysis of principal coordinates (CAP) revealed that frond length was strongly and positively correlated with the abundance of the polychaete *Spirorbis* sp., and that holdfast diameter was positively correlated with the abundance of polychaetes of the Nereididae and Terebelidae family and the limpet *Nacella mytilina* (Figure 5). On the other hand, the abundance of the peracarids *Cassidinopsis emarginata* and *Peramphithoe femorata* was negatively correlated with kelp maximum length and number of stipes. Both species were more abundant during summer months. The CAP ordination accounted for a 64% of the variation in community structure. In addition, we detected a very low probability that this fit was obtained by chance (ANOVA, F_9,68_ = 1.86; *p* = .001).

## 4 DISCUSSION

In this study, we showed that different kelp morphological attributes can have different effects on species richness, total abundance, and the structure of kelp‐associated communities of mobile invertebrates in a subantarctic ecosystem. As expected, a bigger kelp (larger holdfasts, larger maximum length, and greater wet weight) supported more species and more individuals, while the number of stipes exhibited a reversed (albeit minor) effect on these variables. These results emphasize the role of the multiple dimensions of kelp habitat complexity for local biodiversity as a balance between positive and negative effects on associated species.

### 4.1 Positive associations between species richness and abundances with kelp complexity attributes

Holdfast diameter was strongly and positively related to species diversity and abundance in our study. While this result may conform to a specific form of the general species–area relationship (Preston, 1962), part of the pattern may be related to the availability of interstices (Dibble et al., 1996) within the holdfast: Bigger holdfast means a larger number of interstices available for multiple taxa, mainly polychaetes, echinoderms, and molluscs. Terebellid and Nereidid polychaetes showed strong correlations with holdfast diameter in our multivariate analysis (Figure 5), likely due to their ability to use interstices as shelter. Moreover, the positive association between holdfast diameter and the abundance of *Nacella mytilina* could be explained by the consumption of this species on kelp‐associated epibiotic diatoms (Rosenfeld, 2016). Thus, larger holdfasts would provide greater niche opportunities in terms of habitable space and food for associate fauna.

In line with our results, kelp holdfasts have been previously defined as "micro‐ecosystems" (sensu Ortega et al., 2014) that can be used as indicators of kelp forest ecological health (Teagle et al., 2017; Vásquez et al., 2012; Vega, 2016; Villegas et al., 2008). The holdfast structure offers protection from predators and adverse environmental conditions, accumulates food sources, and increases the area and volume of habitable space for colonization (e.g., Christie et al., 2003; Tuya et al., 2011; Vásquez & Santelices, 1984). For example, each holdfast of the kelps *Saccorhiza polyschides* and *Laminaria hyperborean* can harbor more than 400 individuals of amphipods, molluscs, and polychaetes (Tuya et al., 2011). The latter two groups also dominate the holdfast communities of the complex *Lessonia berteroana‐spicata* in Chile (Vega, 2016). Another study in *L. berteroana‐spicata* found 43 species on the kelp holdfasts, over 70% of the individual were juveniles (Cancino & Santelices, 1984). This is consistent with our findings: Some of the species, like *Aulacomya atra,* were found only as juveniles in *L. flavicans* holdfasts. This highlights the importance of this microhabitat for larval recruitment.

Maximum length and wet weight, which represent bigger kelps and more settlement area, had also positive effects on species richness and abundance. The species–area relationship is one of the most general patterns in ecology, and it can be generated by three main mechanisms: an increase in the number of individuals with the increasing area, an increase in the range of ecological conditions, and a reduction in extinction rates (Ben‐Hur & Kadmon, 2020). Greater habitable surface could well explain the positive relationship observed between frond length and the abundance of spirorbid polychaetes, which build permanent shells on the substratum.

### 4.2 Negative diversity‐kelp complexity relationships

Surprisingly, the number of stipes had a negative effect on species richness and total abundance, and kelp frond length negatively affected the abundance of the numerically dominant peracarids *Cassidinopsis emarginata* and *Peramphithoe femorata*. These negative effects could be related to the indirect effects of enhanced shading and altered water flow regimes on juveniles. In addition, thallus scour and whiplash‐like movements of longer fronds could well reduce the abundance of amphipods using these structures for attachment (Kiirikki, 1996; Teagle et al., 2017).

An alternative and nonexclusive explanation to these negative associations would stem from invertebrate's life history traits and behavior. *Peramphithoe femorata* builds nest‐like domiciles by rolling up portions of kelp fronds: A 2‐week experiment conducted in northern‐central Chile demonstrated that, within 8 days, blade elongation of *Macrocystis pyrifera* blades can equal amphipod's nest construction speed; after that moment, nest advancement rate surpasses the frond growth rates, forcing amphipods to migrate to other blades or kelps (Cerda et al., 2010). However, and to our best knowledge, there is no detailed description of amphipod nest‐building behavior in relation to *L. flavicans*' growth rates. Despite this limitation, our study suggests that the role of canopy‐forming species in structuring the local communities could be the result of a balance between positive and negative effects on individual species (see Valdivia et al., 2012 for an example from intertidal communities).

### 4.3 Spatiotemporal variation in kelp‐associated communities

The Magellan region is characterized by strong seasonal changes in abiotic factors, which may significantly influence species richness and biomass (Ojeda et al., 2014). In this study, species richness and total abundance tended to increase in the periods of high productivity (spring and summer), which agrees with previous studies of kelp communities in the Magellan region (e.g., Asensi & Küpper, 2012).

Species richness and abundance gradually varied over the year: The highest species richness and abundance were observed in spring‐summer, and the lower numbers were observed in winter. In this region, the strong seasonal changes in light availability (Palacios et al., 2020) can be reflected into temporal variations in kelp growth rates (Blain & Shears, 2020; Gendron, 1989). Moreover, the numerically dominant amphipods, in addition to *A. atra* and the keyhole limpet *Fissurella picta*, increased in abundance during the spring season. This reflects the importance of seasonality in abiotic environmental conditions for community structure in this region. The detected patterns suggest that the current climate change‐related climatic anomalies in Patagonia (*e.g.,* Garreaud, 2018; Aguayo et al., 2019) could have severe effects on the diversity, structure, and functioning of these communities.

## 5 CONCLUSION

In conclusion, the physical attributes of the kelp *Lessonia flavicans*, a foundation species, were shown to be robust predictors of kelp‐associated biodiversity. Kelp habitat complexity influenced species richness, community abundance, and species‐specific abundances as a balance between positive and negative effects on associated species. This balance, however, tilted toward positive effects of habitat complexity on biodiversity: Overall, bigger kelps supported more speciose and abundant communities. This study highlights the importance of including habitat complexity in future models and criteria for benthic resources management, which will help us to conserve these natural ecosystems that are facing multiple anthropogenic threats.

## CONFLICT OF INTEREST

The authors declare that they have no conflict of interest.

## AUTHOR CONTRIBUTION

**Catalina Velasco‐Charpentier:** Conceptualization (equal); Data curation (lead); Formal analysis (equal); Funding acquisition (equal); Investigation (lead); Methodology (equal); Writing‐original draft (lead). **Felipe Pizarro‐Mora:** Methodology (supporting); Writing‐original draft (supporting). **Nelso P. Navarro:** Conceptualization (equal); Funding acquisition (equal); Resources (equal); Supervision (equal); Writing‐review & editing (equal). **Nelson Valdivia:** Conceptualization (equal); Formal analysis (equal); Funding acquisition (equal); Investigation (equal); Methodology (equal); Resources (equal); Supervision (lead); Writing‐review & editing (lead).

## Supporting information

Fig S1‐S2

## Data Availability

The dataset is available from the Dryad Digital Repository in https://doi.org/10.5061/dryad.qz612jmd6
