# Supplementary material for: Disentangling the links between habitat complexity and biodiversity in a kelp‐dominated subantarctic community
Source: Ecol Evol. 2021 Jan 15;11(3):1214–24. doi: 10.1002/ece3.7100 (PMC7863391; doi:10.1002/ece3.7100)
Supplement: Supplementary file 1 — Fig S1‐S2 [file ECE3-11-1214-s001.docx]

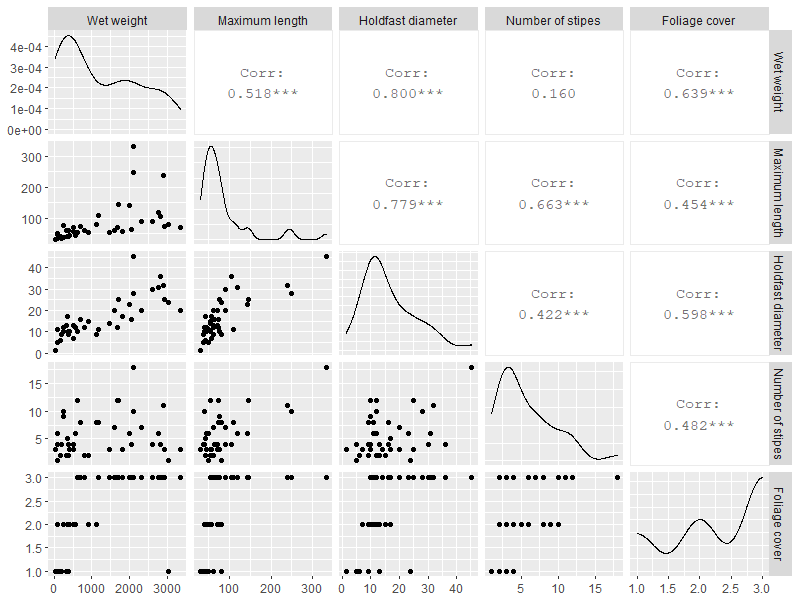


**Fig S1**. Scatterplot matrix (SPLOM) showing Pearson product-moment correlations between kelp habitat complexity dimensions. *** means statistically significant correlations with p < 0.001.

**Fig S2**. GLM Coefficients outputs for species richness and total abundance. Species richness pseudo-R^2^ = 0.51; total abundance pseudo-R^2^ = 0.88. * indicates pairwise contrasts between sites (Carrera and Bahía Buzos) and between each year season and spring.

| Species richness | | | |
| --- | --- | --- | --- |
|  | Estimate | Std. Error | P(>\|z\| \| H0) |
| PC1 | 3.85 | 0.65 | <0.0001 |
| PC2 | 2.39 | 0.56 | <0.0001 |
| *Carrera | -0.30 | 0.14 | 0.03 |
| *Summer | -0.10 | 0.16 | 0.51 |
| *Autumn | -0.61 | 0.22 | 0.007 |
| *Winter | -1.05 | 0.27 | 0.0001 |
| Total abundance | | | |
| PC1 | 5.08 | 0.39 | <0.0001 |
| PC2 | 2.97 | 0.32 | <0.0001 |
| *Carrera | -0.58 | 0.07 | <0.0001 |
| *Summer | -0.50 | 0.08 | <0.0001 |
| *Autumn | -1.36 | 0.12 | <0.0001 |
| *Winter | -1.92 | 0.16 | <0.0001 |
